# Correlates of antenatal care utilization among women of reproductive age in sub-Saharan Africa: evidence from multinomial analysis of demographic and health surveys (2010–2018) from 31 countries

**DOI:** 10.1186/s13690-020-00516-w

**Published:** 2020-12-14

**Authors:** Sulaimon T. Adedokun, Sanni Yaya

**Affiliations:** 1 Department of Demography and Social Statistics, Obafemi Awolowo University, Ile-Ife, Nigeria; 2 School of International Development and Global Studies, Faculty of Social Sciences, University of Ottawa, 120 University Private, Ottawa, ON K1N 6N5 Canada; 3 The George Institute for Global Health, Imperial College London, London, UK

**Keywords:** Antenatal care, Sub-Saharan, Africa, Utilization, Women, Multinomial, Maternal, Service, Facility

## Abstract

**Background:**

Despite a global reduction of about 38% in maternal mortality rate between 2000 and 2017, sub-Saharan Africa is still experiencing high mortality among women. Access to high quality care before, during and after childbirth has been described as one of the effective means of reducing such mortality. In the sub-region, only 52% of women receive at least four antenatal visits. This study examined the factors influencing antenatal care utilization in sub-Saharan Africa.

**Methods:**

Data from Demographic and Health Surveys (DHS) of 31 countries involving 235,207 women age 15–49 years who had given birth to children within 5 years of the surveys were used in the study. Multinomial logistic regression model was applied in the analysis.

**Results:**

About 13% of women in sub-Saharan Africa did not utilize antenatal care while 35 and 53% respectively partially and adequately utilized the service. Adequate utilization of antenatal care was highest among women age 25–34 years (53.9%), with secondary or higher education (71.3%) and from the richest households (54.4%). The odds of adequate antenatal care utilization increased for women who are educated up to secondary or higher education level, from richest households, working, living in urban areas, exposed to media and did not experience problem getting to health facility or obtaining permission to visit health facility.

**Conclusions:**

This study has revealed information not only on women who did not utilize antenatal care but also on women who partially and adequately utilized the service. The study concluded that the correlates of antenatal care utilization in sub-Saharan Africa include socioeconomic and demographic factors, getting permission to visit health facility, unwillingness to visit health facility alone and problem encountered in reaching the health facility.

## Background

Although maternal mortality reduced globally by close to 38% between 2000 and 2017, sub-Saharan Africa is still experiencing high maternal deaths [1]. The sub-region accounted for about two-thirds of maternal deaths worldwide in 2017 [1]. Records indicate that among the 15 countries that were considered as hot spots of maternal mortality, 8 were from sub-Saharan Africa. These countries include Somalia, Central Africa Republic, Democratic Republic of Congo, Chad, Guinea, Zimbabwe, Nigeria and Ethiopia [1]. Maternal mortality has been linked to some major predictors such as haemorrhage, sepsis, eclampsia/preeclampsia, ruptured uterus, prolonged obstructed labour and complications of abortion, among others [2].

Access to high quality care before, during and after childbirth has been identified as one of the effective means of reducing maternal mortality [3]. Emphasis has been placed on the importance of antenatal care to maternal and child survival. Antenatal care helps women to prepare for delivery and understand warning signs during pregnancy and childbirth [4]. In addition, antenatal care can (i) be a source of micronutrient supplementation, treatment of hypertension for eclampsia prevention and immunization to prevent tetanus (ii) provide the opportunity for HIV testing and to receive medications to prevent mother-to-child transmission of HIV in the event of pregnant women being HIV positive (iii) provide the opportunity to receive medications and insecticide-treated mosquito nets in order to prevent malaria [4]. In a bid to ensure adequate care for women during pregnancy, WHO at a time recommended at least four antenatal visits for every pregnant woman which has been reviewed upward to eight visits throughout the period of pregnancy. In 2016, the organization developed and published 39 recommendations which are related to five interventions aimed at ensuring a positive pregnancy experience for women [5]. Such interventions include nutritional interventions, maternal and foetal assessment, preventive measures, interventions for common physiological symptoms and health system interventions to improve utilization and quality of antenatal care [5].

However, antenatal care utilization among women in sub-Saharan Africa has been below the global rate. Evidence shows that globally 86% of pregnant women access antenatal care with a skilled professional at least once while 65% receive at least 4 visits. Only 52% of pregnant women in sub-Saharan Africa receive at least 4 visits [4]. Low utilization of antenatal care has been linked to factors such as unplanned pregnancy, previous pregnancy complications, poor autonomy, lack of husband’s support, increased distance to health facility, not having health insurance and high costs of services [6]. Other factors that influence antenatal care utilization include maternal age, maternal education, place of residence, household wealth, region, exposure to mass media, number of living children, knowledge during danger signs, previous obstetrical history and quality of care, among others [7–12].

Most of these studies did not use a multi-country approach to study antenatal care utilization. Even studies that used multi-country approach either treated the countries individually or dichotomized the outcome variable into utilized and did not utilize. This study aimed at filling these gaps by applying a multinomial logistic regression model to a pooled data set of countries in sub-Saharan Africa in order to examine the factors that influence partial utilization, adequate utilization and non-utilization of antenatal care among women in the sub-region.

## Methods

### Data and sample

This study used data from Demographic and Health Surveys (DHS) of 31 sub-Saharan African countries which were conducted between 2010 and 2018. The surveys are cross-sectional and obtained information on health and other related issues from women of reproductive age (15–49 years). Sample selection in the surveys involved a two-stage stratified sampling method. Each country was divided into clusters. In the first stage, enumeration areas (EAs) were selected in each cluster and a household listing exercise was conducted in in all selected enumeration areas. The list of households was used as a basis for household selection. In the second stage, households were selected from each enumeration area. In each selected household, women within 15–49 years of age who were either permanent residents or visitors in the night preceding the survey were selected and interviewed. Such women were engaged in a face-to-face interview by field workers who recorded the information in questionnaires. Issues covered in each questionnaire included socioeconomic characteristics, reproductive history, antenatal, delivery and postnatal care, breastfeeding, domestic violence, childhood vaccinations and illnesses, among others. In this study, 235,207 women who have had at least one birth within five years preceding the surveys were involved. The number of women involved in the study and the years of surveys for each country are presented in Table 1.

**Table 1:** Year of survey, number of women and antenatal care utilization in Sub-Saharan Africa using Demographic and Health Surveys 2010–2018

Country	Year of survey	Number of women	Antenatal care utilization		
			Not utilized (%)	Partially utilized (%)	Adequately utilized (%)
Angola	2015–2016	8947	22.5	20.6	56.9
Benin	2017–2018	8994	14.0	34.7	51.3
Burkina Faso	2010	10,364	4.6	60.8	34.6
Burundi	2016–2017	8660	0.7	49.8	49.5
Cameroon	2011	7642	13.2	24.2	62.6
Chad	2014–2015	11,081	41.8	30.1	28.1
Comoros	2012	2015	21.2	28.3	50.5
Congo	2011–2012	6463	10.2	16.4	73.4
Cote d’Ivoire	2011–2012	5425	9.6	47.2	43.1
Democratic Republic of Congo	2013–2014	11,228	13.4	41.6	45.0
Ethiopia	2016	7193	34.8	29.0	36.2
Gabon	2012	4143	9.0	22.6	68.4
Gambia	2013	5385	0.8	21.1	78.1
Ghana	2014	4294	3.6	10.4	86.0
Guinea	2018	5530	17.1	48.2	34.7
Kenya	2014	14,945	6.5	39.4	54.1
Lesotho	2014	2596	5.4	20.4	74.2
Liberia	2013	5348	7.6	19.4	73.0
Malawi	2015–2016	13,448	2.1	47.1	50.8
Mali	2018	6368	24.4	33.3	42.3
Namibia	2013	3952	24.7	11.9	63.4
Niger	2012	7680	15.7	51.2	33.1
Nigeria	2018	21,792	26.1	17.4	56.5
Rwanda	2014–2015	5955	0.8	55.1	44.1
Senegal	2010–2011	8147	7.2	47.2	45.6
South Africa	2016	3036	8.1	13.8	78.1
Tanzania	2015–2016	7050	2.2	48.3	49.5
Togo	2013–2014	5016	7.5	37.2	55.3
Uganda	2016	10,263	2.3	37.9	59.8
Zambia	2018	7372	2.1	33.6	64.3
.Zimbabwe	2015	4833	5.7	18.1	76.2

### Outcome variable

Outcome variable in this study is antenatal care utilization which was measured as not utilized, partially utilized and adequately utilized. The ‘not utilized’ category involved women who did not attend antenatal clinic at all when they were pregnant. While the ‘partially utilized’ category included women who attended antenatal clinic less than 4 times, the ‘adequately utilized’ category involved women who attended antenatal clinic 4 or more times during their pregnancy period.

### Independent variables

In this study, the following independent variables were considered: age, education, household wealth, residence, employment, media exposure, parity, getting permission to use health facility, distance to health facility and unwillingness to visit health facility alone. Age was categorized as 15–24 years, 25–34 years and 35 years and above. Education was defined as none, primary and secondary or higher. Household wealth was measured through the ownership of household items such as radio, television, car, bicycle, agricultural land, farm animals and housing characteristics such as toilet facilities, water source, flooring/roofing materials, etc. Households were awarded scores based on the number of items available in the households. The scoring was done using principal component analysis. The result was thereafter expressed in five quintiles namely, poorest, poorer, middle, richer and richest. Residence was grouped into urban and rural. Employment was defined as working for those who engaged in one economic activity or another and not working for those who did not engage in economic activities. Media exposure was categorized as exposed and not exposed. Parity was categorized from 1 to 5 or more. Getting permission to use health facility, distance to health facility and unwillingness to visit health facility alone were measured as problem for women who experienced difficulty in respect of each of the variables and not a problem for those who did not experience any difficulty.

### Statistical analysis

Analysis in this study was carried out in three stages. The first stage involved pooling data sets of the 31 countries together in order to have a single data set for sub-Saharan Africa. To ensure that under-enumeration and over-enumeration in the surveys were adequately adjusted for, a weighting factor **(**$\frac{\boldsymbol{v}\mathbf{005}}{\mathbf{1000000}}$**)** was applied to the data. The data were further defined as survey data using the *svyset* command. In the second stage, Chi-Square test was used to describe the relationships between antenatal care utilization and the independent variables. The third stage involved multivariate analysis where multinomial logistic regression was applied. This is a statistical technique used when the outcome variable has more than two categories. The multinomial logistic regression model is given as:

$$ In\ \left(\frac{\pi \left(Y=j|{x}_1,{x}_2,\dots, {x}_p\right)}{\pi \left(Y=J|{x}_1,{x}_2,\dots, {x_p}_1\right)}\right)={\alpha}_j+{\beta}_{j1}{X}_1+{\beta}_{j2}{X}_2+\dots +{\beta}_{jp}{X}_p $$

Where *j* = 1, 2, …, *J* − 1 (categories of the outcome variable) and *J* is the base outcome; *α*_*j*_ represent the intercepts and *β*_*j*1_, … *β*_*jp*_ represent the logit coefficients; and *X*_1_…*X*_*p*_ represent the independent variable [13].

Base outcome in the analysis is none (women who did not attend antenatal clinic). Odds ratios including their corresponding 95% confidence intervals were thereafter obtained. Three *α* levels (0.05, 0.01 and 0.001) were specified for the interpretation of statistical significance. Stata 14 statistical package was used to perform all the statistical operations.

## Results

### Descriptive statistics

Table 1 shows that the countries with the highest percentage of women who did not utilize antenatal care during their pregnancy period include Chad (41.8%), Ethiopia (34.8%) and Nigeria (26.1%). Countries with the highest proportions of women who partially utilized antenatal care are Burkina Faso (60.8%), Rwanda (55.1%) and Niger (51.2%). With respect to adequate utilization of antenatal care, countries with the highest proportions include Ghana (86%), Gambia (78.1%) and South Africa (78.1%). Results in Table 2 show that about 13% of women in sub-Saharan Africa did not utilize antenatal care, 35% partially utilized antenatal care and 53% adequately utilized antenatal care. Adequate utilization of antenatal care was highest among women age 25–34 years (53.9%), with secondary or higher education (71.3%), from the richest households (68.4%) and living in urban area (65.4%). More than half of working women (54.4%) and those who are exposed to media (59.4%) adequately utilized antenatal care. Majority of women with parity 1 adequately utilized antenatal care. Adequate utilization of antenatal care was predominant among women who declared that they had no problem with respect to getting permission to use health facility (55.3%), reaching the health facility (57.9%) not willing to visit health facility alone (55.5%).

**Table 2:** Relationship between correlates and antenatal care utilization in sub-Saharan Africa using Demographic and Health Surveys 2010–2018

Variable	Antenatal care utilization				
	Not utilized	Partially utilized	Adequately utilized	Total	*p*-value
	N (%)	N (%)	N (%)	N (%)	
	29,892 (12.7)	81,058 (34.5)	124,256 (52.8)	235,207 (100.0)	
Age					
15–24	8389 (11.8)	25,492 (35.9)	37,103 (52.3)	70,984 (100.0)	
25–34	13,186 (12.3)	36,174 (33.8)	57,769 (53.9)	107,129 (100.0)	
35+	8317 (14.6)	19,393 (34.0)	29,384 (51.4)	57,094 (100.0)	< 0.001
Education					
None	20,445 (22.4)	35,831 (39.2)	35,008 (38.4)	91,284 (100.0)	
Primary	5912 (7.5)	30,126 (38.2)	42,915 (54.3)	78,953 (100.0)	
Secondary/higher	3535 (5.4)	15,102 (23.2)	46,333 (71.3)	64,970 (100.0)	< 0.001
Household wealth index					
Poorest	11,989 (20.8)	21,551 (37.3)	24,212 (41.9)	57,752 (100.0)	
Poorer	7291 (14.4)	18,856 (37.1)	24,662 (48.5)	50,809 (100.0)	
Middle	5034 (10.9)	16,656 (36.0)	24,568 (53.1)	46,258 (100.0)	
Richer	3725 (8.8)	13,827 (32.7)	24,763 (58.5)	42,313 (100.0)	
Richest	1853 (4.9)	10,169 (26.7)	26,051 (68.4)	38,073 (100.0)	< 0.001
Residence					
Urban	5302 (7.1)	20,376 (27.5)	48,565 (65.4)	74,243 (100.0)	
Rural	24,590 (15.3)	60,683 (37.7)	75,691 (47.0)	160,964 (100.0)	< 0.001
Employment					
Not working	14,176 (16.6)	28,314 (33.2)	42,803 (50.2)	85,293 (100.0)	
Working	15,089 (10.6)	49,599 (35.0)	77,204 (54.4)	141,892 (100.0)	< 0.001
Media exposure					
Not exposed	18,118 (22.1)	30,527 (37.2)	33,368 (40.7)	82,013 (100.0)	
Exposed	11,652 (7.6)	50,399 (33.0)	90,703 (59.4)	152,754 (100.0)	< 0.001
Parity					
1	4314 (8.9)	15,290 (31.7)	28,669 (59.4)	48,273 (100.0)	
2	4631 (10.6)	14,396 (32.9)	24,724 (56.5)	43,751 (100.0)	
3	4291 (11.6)	12,496 (33.8)	20,143 (54.5)	36,930 (100.0)	
4	3993 (13.2)	10,446 (34.6)	15,751 (52.2)	30,190 (100.0)	
5+	12,663 (16.7)	28,431 (37.4)	34,969 (45.9)	76,063 (100.0)	< 0.001
Getting permission to use health service					
A problem	8265 (20.6)	14,021 (34.9)	17,866 (44.5)	40,152 (100.0)	
Not a problem	17,402 (10.0)	60,604 (34.7)	96,462 (55.3)	174,468 (100.0)	< 0.001
Distance to health facility					
A problem	14,437 (16.4)	32,420 (36.9)	40,961 (46.7)	87,818 (100.0)	
Not a problem	11,235 (8.9)	42,204 (33.2)	73,368 (57.9)	126,807 (100.0)	< 0.001
Not willing to go to health facility alone					
A problem	8970 (18.7)	17,144 (35.7)	21,964 (45.6)	48,078 (100.0)3	
Not a problem	16,698 (10.0)	57,478 (34.5)	92,358 (55.5)	166,534 (100.0)	< 0.001

### Correlates of antenatal care utilization

Results from multinomial logistic regression showing the correlates of antenatal care utilization are presented in Table 3.

**Table 3:** Results of multinomial logistic regression for antenatal care utilization in sub-Saharan Africa using Demographic and Health Surveys 2010–2018

Variable	Antenatal care utilization	
	Partially utilized vs Not utilized	Adequately utilized vs Not utilized
Age	aOR (95% CI)	aOR (95% CI)
15–24	1	1
25–34	0.94* (0.90–0.0.99)	1.23*** (1.18–1.29)
35+	0.84*** (0.79–0.89)	1.29*** (1.22–1.36)
Education		
None	1	1
Primary	2.24*** (2.16–2.32)	3.14*** (3.03–3.25)
Secondary/higher	1.34*** (1.28–1.41)	3.36*** (3.22–3.52)
Household wealth index		
Poorest	1	1
Poorer	1.29*** (1.24–1.34)	1.34*** (1.29–1.39)
Middle	1.61*** (1.54–1.68)	1.64*** (1.57–1.71)
Richer	1.88*** (1.78–1.98)	1.89*** (1.79–1.99)
Richest	2.33*** (2.17–2.49)	2.41*** (2.25–2.58)
Residence		
Urban	0.84*** (0.80–0.88)	1.16*** (1.11–1.20)
Rural	1	1
Employment		
Not working	1	1
Working	1.49*** (1.44–1.53)	1.54*** (1.49–1.58)
Media exposure		
Not exposed	1	1
Exposed	1.78*** (1.72–1.83)	2.11*** (2.05–2.18)
Parity		
1	1.16*** (1.09–1.24)	1.65*** (1.55–1.75)
2	1.04 (0.99–1.10)	1.33*** (1.26–1.40)
3	1.03 (0.98–1.08)	1.23*** (1.17–1.29)
4	1.00 (0.95–1.05)	1.15*** (1.09–1.21)
5+	1	1
Getting permission to use health service		
A problem	1	1
Not a problem	1.59*** (1.53–1.65)	1.73*** (1.67–1.79)
Distance to health facility		
A problem	1	1
Not a problem	1.09*** (1.05–1.13)	1.19*** (1.16–1.24)
Not willing to go to health facility alone		
A problem	1	1
Not a problem	1.26*** (1.21–1.31)	1.27*** (1.22–1.31)

Level of significance at *p < 0.05, **p < 0.01, ***p < 0.001

#### Partial antenatal care utilization

There is a significant relationship between age and partial utilization of antenatal care as women age 25–34 years and 35 years and above are 6 and 16% respectively less likely to utilize antenatal care partially compared to women age 15–24 years. Women with secondary or higher education are 34% more likely to partially utilize antenatal care compared to uneducated women. Household wealth indicates that women from richest households are 133% more likely to partially utilize antenatal care than women from poorest households. However, results from residence show that women living in urban areas are 16% less likely to partially utilize antenatal care compared to rural women. The odds of partial utilization of antenatal care increased by factors of 1.49 and 1.78 respectively for working women and those who have media exposure. Women with parity 1 are 16 times more likely to partially utilize antenatal care compared to women with parity 5 or more. The odds of utilizing antenatal care partially increased by factors of 1.59 and 1.09 respectively for women who did not experience any problem with respect to getting permission to use health facility and reaching the health facility. Women who did not experience any problem in respect of not willing to visit health facility alone are 26% more likely to partially utilize antenatal care compared to women who experienced problems.

#### Adequate antenatal care utilization

Results indicate that women who are within the age groups 25–34 years and 35 years and above are 23 and 29% respectively more likely to adequately utilize antenatal care compared to women of age group 15–24. While the odds of adequate utilization of antenatal care increased by a factor of 3.36 for women with secondary or higher education, the odds increased by a factor of 2.41 for women from richest households. Women who are living in urban areas and those who are working are 16 and 54% respectively more likely to utilize antenatal care adequately. The odds of utilizing antenatal care adequately increased by a factor of 2.11 for women who are exposed to media. Women with parity 1 are 65% more likely to adequately utilize antenatal care compared to women with parity 5 or more. The odds of utilizing antenatal care adequately increased by factors of 1.73 and 1.19 respectively for women who did not experience problems getting permission to use health facility and reaching the health facility. Women who did not experience problems with respect to unwillingness to visit health facility alone are 27% more likely to adequately utilize antenatal care compared to women who experienced problems.

## Discussion

This study has shown that 35% of women in sub-Saharan Africa partially utilized antenatal care while 53% adequately utilized the service. However, 13% of the women did not utilize the service at all. The study also revealed the factors influencing antenatal care utilization. A significant relationship exists between women’s age and adequate utilization of antenatal care [14–16]. The higher the age of a woman, the more the woman would adequately utilize antenatal care. This indicates that young women (15–24 years) probably lack experience in pregnancy care compared to older women. Results also show that women with secondary or higher education are more likely to utilize antenatal care adequately than uneducated women. This implies that the higher the level of education, the more a woman utilizes antenatal care [17–19]. Education promotes a better enlightenment on issues especially health-related ones and this gives women with secondary or higher education an advantage over their uneducated counterparts. Household wealth shows a significant relationship with antenatal care utilization as women from richest households have higher probability of utilizing antenatal care adequately than women from poorest households [20–24]. Poverty at household level may constitute a great barrier to accessing maternal health services. Women from poor households may not have the financial resources needed to either get registered at clinics or pay for the services rendered during the prenatal period. This may lead to a situation where such women would partially attend the clinics or not attend at all. Residence is another factor which influences antenatal care utilization [25–28]. Rate of adequate utilization of antenatal care in urban areas is higher than that of rural areas. It is obvious that infrastructural facilities are well spread in urban areas and this goes for health facilities which are inadequately provided in rural areas. This gives women in urban areas the opportunity to utilize health services. At the same time, women in urban areas have access to information on health-related issues more than women in rural areas. Results further show that the odds of adequate antenatal care utilization is higher among working women than non-working women [29, 30]. This may be linked to the fact that working women, particularly those in the formal sector, have the opportunity of enjoying health insurance scheme which caters for pregnancy care. In some countries, provisions are made to accommodate women working in the informal sector in the scheme. This enhances antenatal care utilization among such category of women compared to non-working women. Media exposure plays an important role in antenatal care utilization as women who are exposed to media have higher percentage of adequate antenatal care utilization than non-exposed women [31–33]. Access to information on health issues is a significant component of maternal health. When women are furnished with adequate information on maternal health services, it tends to increase their utilization of such services. This informed the implementation of programmes promoting maternal health on radio and television. Parity is another factor which is significantly related to adequate utilization of antenatal care [34–36]. Women with 1 parity are more likely to utilize antenatal care adequately than women with 5 or more parity. This means that the more the parity the less the adequate utilization of antenatal care by women. This finding is corroborated by previous studies which emphasized that poor antenatal care utilization among high parity women might be linked to increased confidence from previous pregnancies and childbirth [6, 8, 37–39]. With respect to getting permission from spouse in order to use health service, women who claimed that they did not experience any problem getting permission are more likely to utilize antenatal care adequately than women who experienced problems in that regard [40, 41]. This is an aspect where decision to utilize health service by women is hinged on the consent of their husbands. If such husbands do not see any need for their wives to attend antenatal care, it becomes a problem for such women to access health service as acting against the decision of their husbands may be viewed as an unpardonable act of insubordination. This may eventually lead to a serious marital conflict. More so, rate of adequate utilization of antenatal care is higher among women who claimed that they did not experience any problem in respect of distance covered to access health facility compared to women who complained that they experienced difficulty. Problems in terms of distance could imply that the location of health facility is far from the women’s residence and the means of getting there is problematic [39, 42–44]. For instance, women living in areas where there is poor road network such that vehicles hardly ply the road or areas where transport system is irregular would find it difficult access health facility. This shows that even when such women are willing to utilize antenatal care, their inability to navigate easily to the facility will prevent them from making use of such service. Unwillingness to visit health facility alone is another factor determining adequate antenatal care utilization. In some cases, women may not be willing to visit health facility without being accompanied by either their husbands or close relatives based on their beliefs or conviction [45]. Women who are of the opinion that unwillingness to visit health facility alone is not their problem are more likely to utilize antenatal care adequately than women who claimed that it is their problem. This implies that the more the number of women who consider not visiting health facility alone, the less the proportions of women that would adequately utilize antenatal care.

### Policy implications

With 13% of women not utilizing antenatal care, 35% partially utilizing it and 53% adequately utilizing it, antenatal care utilization in sub-Saharan Africa remains a big health challenge. This may impede the efforts at reducing maternal mortality in the region. It is apparent that more efforts need to be made in order to increase the proportions of women that would adequately utilize antenatal care. One of the ways of achieving this is through provision of more health facilities particularly in areas where they are grossly inadequate. Rural areas should be considered in this regard. There is a need to increase awareness on importance of antenatal care utilization among young woman who are just beginning their childbearing experience. Such awareness initiatives should be organized in both rural and urban areas and emphasis should be placed on the risks involved in not attending antenatal care at all or partially attending it. It is necessary to propagate such awareness on radio and television programmes. Efforts should also be made towards increasing proportions of educated women in each country. Incentives that would encourage enrolment of female children in school should be introduced. Poverty alleviation initiatives both at individual and household levels through social security, employment opportunities and funds for business activities should be pursued. It is also necessary for governments at different levels to introduce initiatives that would actively involve men in maternal health care. This is important in order to address the problems associated with husbands not granting their wives permission to visit health facility or not willing to accompany them to utilize health service. Involving them in maternal health care would improve their awareness on adequate antenatal care utilization which would make them support their wives during and after pregnancy. This initiative should be undertaken at local and national levels and should be part of maternal health care agenda which would be supervised by Ministry of Health in each country.

### Strengths and weaknesses

This study is based on data sets obtained from cross-sectional surveys which were conducted over some periods of time. This has only provided the opportunity to arrive at associated factors of antenatal care utilization but not the causes. Because the surveys were conducted at different periods, the variations in the characteristics of the respondents which occurred over these periods among the countries could not be harmonized. The analysis could not also include the time and countries as variables. It should also be noted that responses in the surveys were obtained from participants who reported events that had occurred in the past. Such responses may be fraught with recall error. In spite of these weaknesses, the study has examined the correlates of antenatal care utilization by applying multinomial logistic regression model which disaggregated the outcome variable into three categories. This provided an in-depth explanation on the effects of the correlates on each category. This is in contrast to most previous studies that modelled the outcome variable as binary. Moreover, the study used large data sets from 31 countries in sub-Saharan Africa which made the findings to be robust and generalizable.

## Conclusions

The study has revealed information not only on women who did not utilize antenatal care in sub-Saharan Africa, but also those who partially or adequately utilized it. The study has also shown that apart from socioeconomic and demographic factors, other factors that influence antenatal care utilization include getting permission to visit health facility, unwillingness to visit health facility alone and distance to health facility. There is a need to address the poor utilization of antenatal care by developing a maternal health service utilization model which would consider the different factors.

## Data Availability

Data used in this study were obtained from the DHS Program and available at: https://dhsprogram.com/data/available-datasets.cfm.

## Abbreviations

aOR: Adjusted Odds Ratio
CI: Confidence Interval
DHS: Demographic and Health Survey
UNICEF: United Nations International Children’s Emergency Fund
WHO: World Health Organization
